# Extracellular matrix metalloproteinase inducer in brain ischemia and intracerebral hemorrhage

**DOI:** 10.3389/fimmu.2022.986469

**Published:** 2022-08-31

**Authors:** Yang Liu, Yanling Mu, Zhe Li, Voon Wee Yong, Mengzhou Xue

**Affiliations:** ^1^ Department of Cerebrovascular Diseases, The Second Affiliated Hospital of Zhengzhou University, Zhengzhou, China; ^2^ Henan International Joint Laboratory of Intracerebral Hemorrhage and Brain Injury, The Second Affiliated Hospital of Zhengzhou University, Zhengzhou, China; ^3^ Hotchkiss Brain Institute and Department of Clinical Neurosciences, University of Calgary, Calgary, AB, Canada

**Keywords:** Extracellular matrix metalloproteinase inducer (EMMPRIN), brain ischemia, intracerebral hemorrhage, matrix metalloproteinases, neuroinflammation

## Abstract

Increasing evidence from preclinical and clinical studies link neuroinflammation to secondary brain injury after stroke, which includes brain ischemia and intracerebral hemorrhage (ICH). Extracellular matrix metalloproteinase inducer (EMMPRIN), a cell surface transmembrane protein, is a key factor in neuroinflammation. It is widely elevated in several cell types after stroke. The increased EMMPRIN appears to regulate the expression of matrix metalloproteinases (MMPs) and exacerbate the pathology of stroke-induced blood-brain barrier dysfunction, microvascular thrombosis and neuroinflammation. In light of the neurological effects of EMMPRIN, we present in this review the complex network of roles that EMMPRIN has in brain ischemia and ICH. We first introduce the structural features and biological roles of EMMPRIN, followed by a description of the increased expression of EMMPRIN in brain ischemia and ICH. Next, we discuss the pathophysiological roles of EMMPRIN in brain ischemia and ICH. In addition, we summarize several important treatments for stroke that target the EMMPRIN signaling pathway. Finally, we suggest that EMMPRIN may have prospects as a biomarker of stroke injury. Overall, this review collates experimental and clinical evidence of the role of EMMPRIN in stroke and provides insights into its pathological mechanisms.

## Introduction

Stroke is the second leading cause of death worldwide, accounting for ~11% of the total number of deaths per year (1). It has a high morbidity and mortality rate (2, 3). Stroke can be traditionally classified into two major categories: ischemic stroke or brain ischemia, and hemorrhagic stroke or intracerebral hemorrhage (ICH). The latter is subdivided into primary intracerebral hemorrhage and subarachnoid hemorrhage (SAH) (4). Ischemic stroke is the sudden interruption of blood circulation in one or more areas of the brain, resulting in hypoxia, inflammation, edema, and accumulation of toxic substances (5). ICH is a sudden rupture of cerebral blood vessels and bleeding into the brain parenchyma. The sustained bleeding from a hemorrhagic stroke leads to mechanical damage resulting from the formation of a growing hematoma and secondary neuroinflammation (6). SAH is a medical emergency that is associated with high mortality and severe disability, although it can be detected and treated early. SAH is caused by blood leaking into the subarachnoid space, usually as a result of ruptured aneurysm and vascular malformation (7).

To date, many patients who survive a stroke cannot live on their own and are at high risk of developing additional neurological sequelae (8, 9). In clinical treatment for acute ischemic stroke, intravenous thrombolysis with recombinant tissue plasminogen activator (rtPA) is the only FDA-approved treatment that limits dying cells around the ischemic penumbra, but it must be administered within 4.5 hours of symptom onset to be effective (10, 11). Due to this narrow treatment window and potential for serious adverse events, such as hemorrhagic transformation (HT) and malignant brain edema associated with the non-thrombolytic effect of rtPA, only < 5% of ischemic stroke patients benefit from rtPA treatment (12). Therefore, contraindications such as borderline coagulation status and large vessel occlusion (LVO) increases the risk of ICH, and often requires patients to undergo mechanical thrombectomy (13).

To prevent hemorrhagic stroke, approaches include the control of blood pressure and other risk factors. To treat hemorrhagic strokes, hemostasis and surgical approaches are the main procedure although they do not significantly improve clinical outcomes (14, 15). Furthermore, potential neuroprotective drugs have failed in clinical trials (16). It is urgent to develop effective methods to treat hemorrhagic as well as ischemic strokes.

Stroke leads to a series of complex pathologic processes, including decreased cerebral perfusion pressure and cerebral blood flow, damage to the blood-brain barrier (BBB), neuronal apoptosis and acute cerebral vasospasm (17, 18). Although the primary injury mechanisms are different between ischemic and hemorrhagic strokes, the injury to neural cells and release of damage-associated molecular patterns (DAMPs) are common pathways that promotes innate and adaptive immune response within the brain and in the periphery (19, 20).

Several studies have emphasized that neuroinflammation is important in the secondary injury following stroke (21–24). Post-stroke neuroinflammation comprises the infiltration of leukocytes from the circulation into the brain, the elevated density and activity of CNS-intrinsic microglia, and the upregulation of inflammatory cytokines, chemokines, free radicals, adhesion molecules and matrix metalloproteinases (MMPs); collectively, these promote the massive injury and loss of neurons (25–27).

Extracellular matrix metalloproteinase inducer (EMMPRIN), also known as cluster of differentiation 147 (CD147), is a type-I highly glycosylated transmembrane protein of the immunoglobulin superfamily (28). EMMPRIN was described 38 years ago (29). EMMPRIN is common to many species and is known by different names, such as basigin (basic immunoglobulin superfamily); gp42 (30) or tumor cell collagenase-stimulatory factor (TCSF) in mice (31); OX47 antigen and CE9 in rats (32); M6 or Hab18G in humans (33, 34); and 5A11, HT7 or neurothelin in chickens (35–37). It is broadly expressed on the surface of various cell types involved in stroke-induced neuroinflammation, including endothelial cells, astrocytes, microglia, leukocytes and platelets (38, 39). Biswas, who initially discovered EMMPRIN, found that MMP-1 was induced by EMMPRIN during the co-culture of tumor cells and fibroblasts (29, 40, 41). Using a transient middle cerebral artery occlusion (tMCAO) in mice, the authors found that inhibition of EMMPRIN reduced infarct size and improved functional outcomes (38). We have described that minocycline exhibits neuroprotective roles in ICH by decreasing EMMPRIN and MMP-9 expression (42).

In the SAH model in rat, the increased expression of EMMPRIN may be an important factor in the formation of brain edema (43). More recent reports have found that the expression of EMMPRIN is increased and plays an important role in the pathological process following ischemia stroke (38, 44) and ICH (42, 43). Thus, EMMPRIN may be a new therapeutic target in stroke.

In this review, we describe and discuss the roles of EMMPRIN in brain ischemia and ICH. We first introduce basic knowledge about EMMPRIN and summarize its functions. Next, we describe the activation of EMMPRIN following brain ischemia and ICH. We then discuss the pathophysiological roles of EMMPRIN in brain ischemia and ICH, and the potential benefits of inhibiting EMMPRIN in stroke.

## Structure of EMMPRIN

EMMPRIN is encoded by the BSG gene, which is located on human chromosome 19 (p13.3) and consists of 10 exons with a span of about 12 kb (45). Four isotypes of EMMPRIN have been identified according to the splicing and variation of transcription start sites (EMMPRIN-1, -2, -3 and -4) (46). EMMPRIN is also divided into highly glycosylated EMMPRIN (HG-EMMPRIN) of molecular weights between ~45 - 65 kDa, and low glycosylated EMMPRIN (LG-EMMPRIN) (~32 kDa) (28). All four isoforms of EMMPRIN are known to be glycosylated (46). The full-length human EMMPRIN is composed of 269 amino acid residues (47), contains a signal peptide (21 amino acid residues), an extracellular domain (185 amino acid residues), a transmembrane domain (24 amino acid residues), and a cytoplasmic domain (39 amino acid residues) (41). Each domain of EMMPRIN can interact with different proteins, and exhibits different functions. The N-terminal extracellular domain consists of two regions characteristic of the immunoglobulin superfamily that are heavily glycosylated (48, 49), and the first Ig domain of EMMPRIN (ECI) has counterreceptor binding activity (50) and is involved in MMP induction (41, 51). Inhibitors of EMMPRIN homophilic interactions such as bivalent CD147-Fc protein and monoclonal antibody prevent MMP production and MMP-dependent invasion of cells through basement membranes (52). In addition, the ECI domain interacts with α3β1 and α6β1 integrins, activating the FAK-PI3K-Ca^2+^ pathway downstream, and affecting the migration of inflammatory cells (53, 54).

The second Ig domain of EMMPRIN (ECII) associates with caveolin-1 and cyclophilins A and B; caveolin-1 has a negative regulatory effect on EMMPRIN self-association and MMP-inducing activity (52). Cyclophilins A and B may engage pathways for survival (55) and chemotaxis (56). EMMPRIN binds to Cyclophilin A to induce MMP-9 production (57). The transmembrane region of EMMPRIN contains a central location of glutamate which is essential for its lateral binding to the monocarboxylic acid transporters MCT1 and MCT4, thereby facilitating proper expression of MCT1 and MCT4 at the cell surface (58, 59). The transmembrane domain also interacts with proteins such as Cyp60 (60).

EMMPRIN is expressed at varying levels widely such as in immune cells, epithelial and endothelial cells (ECs), and tumor cells (32), In the normal mammalian CNS, EMMPRIN is expressed on endothelial cells of the blood-brain barrier, and in other subregions such as the septum, amygdala, thalamic anterior nuclei, hypothalamus, mesencephalic tegmentum, entorhinal cortex, and cingulate gyrus (28, 39, 61). EMMPRIN is expressed in low quantity in normal blood vessels, and is increased in inflammation. Under many pathological conditions, EMMPRIN is highly up-regulated in infiltrated neutrophils, T and B lymphocytes, monocytes, microglia/macrophages, endothelial cells and dendritic cells (38, 39, 42, 62).

Much of what is known about the functions of EMMPRIN comes from studies in EMMPRIN knockout mice (63, 64). It is estimated that only about 30% of EMMPRIN null mice are born, and 50% of the surviving mice die of interstitial pneumonia in the first week after birth (65). EMMPRIN deficient female mice are infertile due to the failure of female reproductive processes including implantation and fertilization (64, 65). EMMPRIN null mice that survive to adulthood exhibit a variety of neurobehavioral disorders, such as reduced awareness to certain smells (63), poor learning and memory (66, 67), and increased sensitivity to electric shocks to the feet (63). In addition, implantation defects may display misregulation of MMP production (68). EMMPRIN null mice also have reduced integrity of the blood-brain barrier (63) and defects of cell cycle in lymphocytes (69).

## Elevation of EMMPRIN following brain ischemia and ICH

### Brain ischemia

Several studies have documented that the expression of EMMPRIN is increased after brain ischemia, likely the result of inflammatory factors (including cytokines, free radicals, and oxidized low-density lipoproteins) (38, 70, 71). The study found that increased EMMPRIN expression may be related to the NF-kappaB pathway after stroke (38). Subsequent pathogenic effects are thought to be related to the induction of MMPs or to the capacity of EMMPRIN to affect chemotaxis through extracellular cyclophilins (38, 72). The activity of EMMPRIN thought to be important in stroke includes EMMPRIN‐mediated induction of MMPs that promotes BBB breakdown and brain injury (73).

One study described that expression of EMMPRIN was elevated in the basal ganglia and cortex of experimental rat models of brain ischemia, where its level was significantly associated with increased MMP expression (70). In a mouse model of permanent focal cerebral ischemia, expression of EMMPRIN and associated MMP-9 was upregulated in the peri-infarct region 2-7 days after ischemia compared to the contralateral non-ischemic hemisphere (71). To investigate the interaction between EMMPRIN and inflammation after brain ischemia, one group used anti-CD147 to block EMMPRIN; the results show that inhibition of EMMPRIN ameliorated acute ischemic stroke by reducing neuroinflammation (38, 44). Clinically, patients with high serum level of EMMPRIN at 24 hours after stroke have poor outcomes even at 12 months after the event (44).

Anti-CD147 function blocking antibody (αCD147) therapy has been found not only to prevent neuronal and oligodendrocyte death in the acute phase after ischemic stroke, but also to profoundly protect white matter integrity and reduce brain atrophy and tissue loss in later stages (74). EMMPRIN has been reported to induce the expression of vascular endothelial growth factor (VEGF) and promote angiogenesis. Angiogenesis is a critical component of neurovascular remodeling following stroke (71).

### Intracerebral hemorrhage

**Figure 1:**
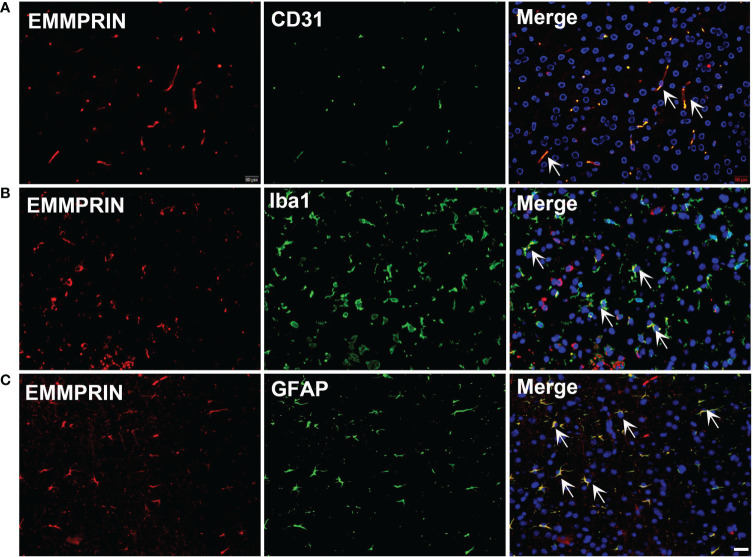
ICH increases the expression of EMMPRIN in astrocytes, microglia and endothelial cells. Representative images of the co-expression of EMMPRIN with CD31 (**A**, endothelial cell marker), Iba1 (**B**, microglia/macrophages), and GFAP (**C**, astrocytes) in mouse brain sections at 3d post-ICH. Images are acquired in the perihematoma region. Scale bar = 20 μm.

EMMPRIN is also elevated following ICH. An earlier study in an experimental subarachnoid hemorrhage (SAH) model used western blot and PCR to determine EMMPRIN upregulation at 24h; this was associated with the formation of brain edema (43). In a recent study, CypA-mediated detrimental effects on pericytes and BBB disruption after SAH was thought to be mediated by EMMPRIN/NF-κB/MMP9 axis, and degradation of junction proteins in the brain (75). In our study, we found that EMMPRIN expression was co-localized with CD31 (endothelial cell), Iba1 (microglia/macrophages) and GFAP (astrocytes) at 3d time point post-ICH (**Figure 1**). EMMPRIN mediated the upregulation of MMP-9 and exacerbated neurological dysfunction in a mouse model of experimental ICH (76). Minocycline conferred neuroprotection in ICH associated with decreased EMMPRIN and MMP-9 expression, alleviation of BBB disruption, reduced neuroinflammation, and lower neuronal degeneration and death (42). Taken together, these studies suggest that EMMPRIN elevation drives the pathologic process of ICH.

## Pathophysiological roles of EMMPRIN in brain ischemia and ICH

**Figure 2:**
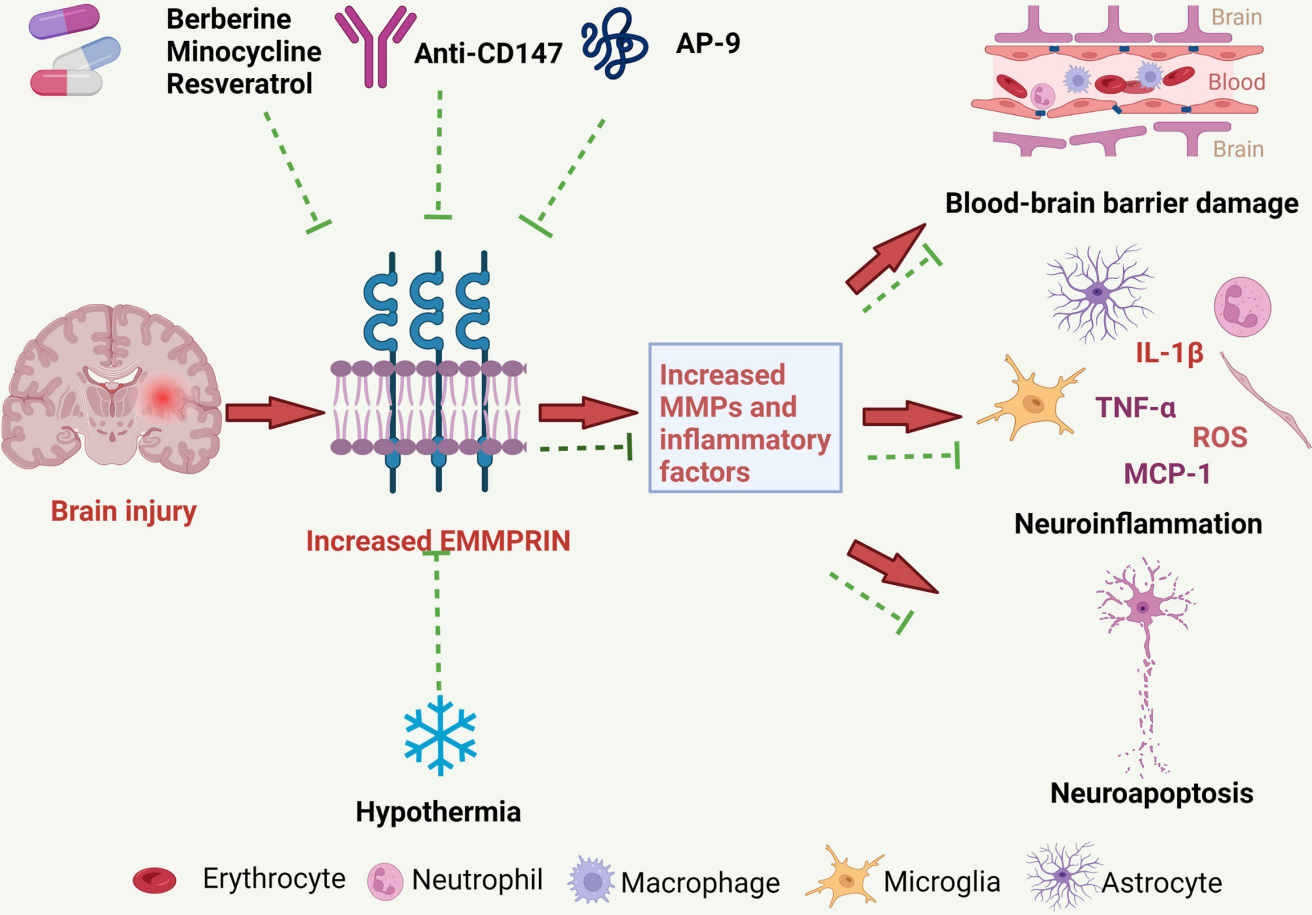
The pathological roles of EMMPRIN in ischemic and hemorrhagic strokes, and treatment strategies. EMMPRIN is elevated in endothelial cells, microglia and astrocytes following stroke. EMMPRIN induces the production of MMPs, and MMPs then degrade extracellular matrix proteins resulting in increased BBB permeability. EMMPRIN can also promote leukocyte extravasation through blood vessels, which further aggravates neuroinflammation. EMMPRIN may promote neuroapoptosis and exacerbate brain damage. Several promising therapies for ischemic and hemorrhagic strokes may target EMMPRIN, and these include medications and physical therapy.

Increased EMMPRIN expression may promote the development of brain injury following brain ischemia and ICH through several mechanisms, to be described further below (**Figure 2**).

### Neuroinflammation

Brain damage after stroke is initiated by disruption of the blood supply in ischemic stroke, or by rupture of the intracerebral or subarachnoid vessels in hemorrhagic strokes. The inflammatory response following stroke is a major factor in the progression of the disease, with neuroinflammation contributed by activation of microglia, infiltration of neutrophils and monocyte-derived macrophages, and the release of cytokines, chemokines, MMPs and other factors from these cells (77). Although inflammation of the central nervous system (CNS) is necessary to protect the brain such as from pathogens, extensive neuroinflammation is detrimental for the CNS (78). EMMPRIN is an effective upstream inducer of the expression of various MMPs and EMMPRIN/MMP signaling is exaggerated in immune cells during neuroinflammation in stroke. A study on tMCAO showed that anti-CD147 treatment almost completely abrogated leukocyte adhesion on brain microvasculature at 24h, thereby reducing subsequent influx of immune cells into the brain parenchyma after acute ischemic stroke (38).

Recent studies have also described that the inflammatory response of the spleen after brain ischemia may be associated with secondary brain injury. Splenic EMMPRIN expression was rapidly upregulated at 4h and 24h after brain ischemia (79). Administration of anti-CD147 inhibited inflammatory cytokine (TNFα, IL-6, IL-1β) and monocyte chemotactic protein-1 (MCP-1, CCL2) expression in the spleen, and this was associated with reduced brain injury in cerebral ischemia (79).

### Blood-brain barrier

Blood-brain barrier (BBB) disruption is a hallmark of ICH. This disruption leads to local vasogenic edema, influx of leukocytes and potentially neuroactive agents into the brain around the hematoma, all of which contribute to brain injury (80). A range of factors have been associated with disruption of BBB following ICH, including inflammatory mediators, MMPs, thrombin, hemoglobin breakdown products, oxidative stress and complement. MMP-9 is known to promote BBB disruption in brain ischemia and ICH, with MMP expression correlating with stroke severity (81). In our previous study in an ICH model, minocycline treatment decreased EMMPRIN and MMP-9 expression, reduced the degradation of tight junction proteins (ZO-1 and occludin), and alleviated BBB disruption (42). Pan and colleagues reported that a CD147/NF-κB/MMP9 axis was activated by cyclophilin A production that occurred after SAH, resulting in pericyte dysregulation and BBB destruction (75). In this context, one study showed that the increased expression of CD147 and activity of MMP-9 affected the integrity of BBB, leading to the migration of leukocytes and brain injury during hemorrhagic transformation (38, 44).

### Neuronal apoptosis

Brain damage from brain ischemia includes neuronal apoptosis, a process of programmed cell death that occurs in multicellular organisms. Studies have noted that therapies targeting EMMPRIN can increase neuronal survival in animal models of brain ischemia. For example, treatment with an anti-EMMPRIN antibody in brain ischemia in mice reduced MMP expression in the perivascular cuff and decreased neuronal injury (38, 44, 82). In one study, application of anti-CD147 not only prevented the death of neurons and oligodendrocytes in the acute phase of brain ischemia, but also robustly protected the integrity of white matter, and reduced brain atrophy and tissue loss in the late stage of ICH (74). Thus, inhibition of EMMPRIN may reduce neuronal apoptosis although it is unclear as to the mechanisms involved.

### Metabolism

Monocarboxylic acids have important metabolic functions in all cells. EMMPRIN acts as a chaperone for the proper folding, translocation and expression of MCT1 and MCT4 on the surface of cells (59). Astrocytes are a major source of lactic acid, a byproduct of glycolysis and a major energy source for neurons (83). The monocarboxylate transporter protein MCT-1 is expressed on astrocytes. EMMPRIN has been shown to be crucial in the proper transport of lactate *via* MCT-1 by astrocytes. In a model of multiple sclerosis, MCT-1 transported lactate from cells to the extracellular environment, where lactate provided energy to neighboring cells such as neurons (59). EMMPRIN-deficient mice exhibit deficits in learning and sensory functions, and have photoreceptor degeneration and memory loss, possibly due to impaired lactate shuttling caused by non-functional MCT (66, 84, 85). Thus, EMMPRIN is essential for normal brain function through its ability to regulate metabolic processes within the brain. The relationship between EMMPRIN and metabolism would need to be further characterized in stroke in the future.

## Treatments that target EMMPRIN in brain ischemia and ICH

While new treatment methods of thrombolysis and mechanical thrombectomy are used in stroke, treatment remains suboptimal. Novel treatments and molecular biomarkers that inform on treatment success still need to be developed. Targeting EMMPRIN may be a prospective therapy for brain ischemia and ICH

### Anti-CD147 monoclonal antibody

Function blocking antibodies to EMMPRIN have been described and may inhibit cyclophilin-related signaling or MMP activity, thereby reducing leukocyte infiltration and tissue destruction. In a mouse model of tMCAO, treatment with an anti-CD147 monoclonal antibody significantly reduced brain inflammatory cell infiltration and ameliorated brain injury (38, 44). Another study reported that application of an anti-CD147 monoclonal antibody reduced neuronal and oligodendrocyte cell death, and led to improved sensorimotor and cognitive functions (74). In a SAH model, an anti-CD147 function blocking antibody ameliorated edema and SAH disease severity (43).

### Antagonistic peptide-9

Antagonistic peptide-9 (AP9) to CD147 is composed of 12 amino acid residues; it inhibits the dimerization of EMMPRIN and EMMPRIN-cyclophilin A interaction (86), thereby reducing MMP expression. AP9 can target EMMPRIN to prevent cancer invasion and metastasis (87, 88). A recent study showed that AP9, likely through inhibition of EMMPRIN-mediated MMP-2/-9 activation and ECM degradation, attenuates ischemia/reperfusion injury in an experimental model of acute myocardial infarction (89), and inhibits MMP-9 and MMP-3 in the ischemic brain and plasma (90).

### Berberine

Berberine is a natural compound extracted from the herbs Cortex phellodendri *(H*uang Bai), *Hydrastis canadensis and Rhizoma coptidis* (Huanglian). For centuries, berberine has been widely used in Chinese and Korean medicine to treat diarrhea and gastrointestinal disorders (91). Recently, numerous studies have found that berberine has a multitude of effects in the treatment of several diseases. For example, berberine reduces plasma glucose and cholesterol levels in cardiovascular disease (92, 93). In addition, berberine inhibits excessive autophagy, likely contributing to reduced myocardial ischemia-reperfusion injury in cardiac myocytes (94). Moreover, in an oxidized low-density lipoprotein (oxLDL)-induced macrophage model, berberine upregulated miR-150-5p level, subsequently inhibiting P2X7 receptor-mediated EMMPRIN and MMP-9 expression by suppressing AMPK-α and MAPK signaling, and exerting anti-atherogenic effects (95). Berberine has also been reported to have neuroprotective effects on ischemic brain injury by lowering intracellular ROS level, inhibiting cellular apoptosis (96), and promoting angiogenesis *via* AMPK-dependent microglial polarization to a homeostatic state following tMCAO (97). In summary, berberine inhibits the expression of EMMPRIN and mediates neuroprotective effects in models of neurological diseases.

### Minocycline

Minocycline is a semi-synthetic second-generation tetracycline derivative with anti-inflammatory and bactericidal effects, and which penetrates the blood-brain-barrier (77, 98). A number of studies have identified minocycline to counteract pathological aspects of ICH, including inhibitory activity against pro-inflammatory microglia/macrophages and MMPs, reducing death signaling in neurons, inhibiting leucocyte migration and antioxidant capacity (99–101). Yong and colleagues (102) found that attenuating the elevation of EMMPRIN on T cells and in EAE mice reduced EAE severity. In our studies, we found that minocycline reduced expression of EMMPRIN and MMP-9, alleviated BBB disruption, and improved functional recovery in the acute phase of ICH in mice (42). These results suggest that minocycline may alleviate brain injury by inhibiting the expression of EMMPRIN.

### Resveratrol

Resveratrol is a non-flavonoid polyphenolic compound that is a major component of many herbal medicines. It has neuroprotective effects in a variety of models of neurological disorders including ischemic stroke, Alzheimer’s disease and Parkinson’s disease (103). In recent years, many studies have shown that resveratrol inhibits the activation of microglia and the expression of inflammatory factors and reduces neuronal apoptosis, thus ameliorating hematoma volume and improving outcomes in an ICH model (104, 105). Ge et al. (106) found that resveratrol significantly down-regulated EMMPRIN expression and MMP-9 production in macrophages *via* PPARγ activation. Importantly, this study suggests that EMMPRIN may be a prominent mechanism by which resveratrol inhibits MMP-9 production in macrophages.

### Chlorogenic acid (CGA, 3-O-caffeoylquinic acid)

CGA is a phenolic compound derived from herbs. It has several biological activities such as anti-inflammatory and anti-oxidant actions (107). It has been reported to cross the BBB to affect the CNS (108). CGA targets Reactive nitrogen species (RNS)/Caveolin-1/MMP signaling pathways that may protect the brain from ischemic stroke. In a rat model of transient middle cerebral artery occlusion, CGA inhibited MMP-2/9 activity, alleviated BBB disruption and reduced brain edema and brain infarction (109). Thus, we investigated the effect of CGA treatment on EMMPRIN. The results show that CGA ameliorated injury after ICH associated with the reduced expression of EMMPRIN and MMP-2/9 (110).

### Hypothermia

Hypothermia is considered to be nature’s “gold standard” for neuroprotection (111). In stroke treatment, hypothermia minimizes secondary brain damage, correlated with leukopenia, and impaired leukocyte migration and phagocytosis. Burggraf et al. (70) showed that EMMPRIN expression was reduced in ischemic brain tissue at low temperature, associated with microvascular protection and smaller focal brain injury; this suggests that inhibiting EMMPRIN is potential treatment for brain ischemia.

## Potential of EMMPRIN as a biomarker

Despite the new methods of thrombolysis and mechanical thrombectomy, the current treatment of stroke is not optimal. Novel biomarkers that are easy to detect and are suitable for a clinical setting should help improve stroke management. Currently, EMMPRIN is considered to be a likely major regulator of BBB integrity after stroke, contributing to MMP-9 mediated BBB breakdown and recruitment of peripheral leukocytes into the CNS. Preclinical studies have found that the expression of EMMPRIN in stroke tissue increased significantly, which prompted the secretion of MMPs, associated with neuroinflammation, cognitive impairment and endothelial dysfunction; these results highlight that EMMPRIN is consequential to ischemic and hemorrhagic stroke (38, 43, 44, 79, 112). In clinical studies, the incidence of intracranial atherosclerosis occlusive lesions in patients with ischemic stroke is extremely high. EMMPRIN is an important inflammatory indicator of atherosclerosis and related diseases, such as ischemic stroke; there is an evidence that EMMPRIN stimulates extracellular matrix degradation and promotes cell migration (52), which is a pathologic mechanism of atherosclerosis. Patrizz et al. (44) reported that serum EMMPRIN was elevated 24h after stroke, and that the level of serum EMMPRIN was related to the prognosis of stroke patients. The increase in EMMPRIN level may cause atherosclerotic plaques to detach and form thrombus. Overall, clinical and experimental research data indicate that blood EMMPRIN levels might act as an important biomarker for stroke.

## Future directions

Although many studies have elucidated the specific underlying mechanisms of stroke and the role of EMMPRIN in brain ischemia and ICH, the development of successful therapies targeting EMMPRIN is still in its infancy, and future studies are necessary. While studies have clarified that the downstream consequences of EMMPRIN activity include the elevation of MMPs, the upstream activators of EMMPRIN have not been clarified. Thus, a major question is what triggers EMMPRIN activation. Furthermore, affecting signaling pathway(s) downstream of EMMPRIN activation may be a promising therapeutic avenue. Another future prospect is the discovery of specific inhibitors of EMMPRIN. The developing field of nanomaterials is increasingly integrated into various fields of medicine and is showing promise (113). The ability to deliver drugs more efficiently than traditional methods is the highlight of the use of nanoparticles, and such nanoparticle-targeted therapy to EMMPRIN may reduce the potential side effects associated with systemic therapy. Currently we are doing relevant work in this area.

## Conclusion

In this review, we have considered the underlying mechanisms at the onset of stroke so as to provide guides for the development of new treatment strategies. We implicate EMMPRIN in the pathophysiological processes associated with increased leukocyte recruitment and MMP induction. In order to improve the management of stroke patients, we propose that the malignant chain reaction regulated by EMMPRIN and its interacting molecules (e.g. cyclophilins, caveolins and integrins described in Section 2), and induction of potentially toxic MMPs, must be inhibited. In addition to the contribution of EMMPRIN to the mechanistic details of the disease process, plasma EMMPRIN levels may be informative as a biomarker for stroke patients, so that effective medications for these conditions can be promptly initiated. In-depth research on these issues should open up new methods for targeted therapy of stroke.

## Author contributions

All authors listed have made a substantial, direct, and intellectual contribution to the work and approved it for publication.

## Funding

The authors acknowledge operating grant support from National Key Research and Development Program of China (grant no: 2018YFC1312200), the National Natural Science Foundation of China (grants no: 82071331, 81870942, and 81520108011), and from the Canadian Institutes of Health Sciences (VY).

## Conflict of interest

The authors declare that the research was conducted in the absence of any commercial or financial relationships that could be construed as a potential conflict of interest.

## Publisher’s note

All claims expressed in this article are solely those of the authors and do not necessarily represent those of their affiliated organizations, or those of the publisher, the editors and the reviewers. Any product that may be evaluated in this article, or claim that may be made by its manufacturer, is not guaranteed or endorsed by the publisher.
